# Very low-grade albuminuria is linked to arterial stiffness in a population-based cohort

**DOI:** 10.1016/j.ajpc.2026.101495

**Published:** 2026-02-21

**Authors:** Victor Walendy, Michael Gekle, Claudia Grossmann, Alexander Kluttig, Frank Bernhard Kraus, Beatrice Ludwig-Kraus, Rafael Mikolajczyk, Thomas Schmid, Oliver Thews, Andreas Wienke, Melanie Zinkhan, Matthias Girndt

**Affiliations:** a Martin-Luther-University Halle-Wittenberg, Department of Internal Medicine II, Halle, Germany; b Martin-Luther-University Halle-Wittenberg, Julius-Bernstein-Institute of Physiology, Halle, Germany; c Martin-Luther-University Halle-Wittenberg, NAKO study cohort recruiting center, Halle, Germany; d University Hospital Halle (Saale), Department of Laboratory Medicine, Unit II Laboratory Medicine and Clinical Chemistry, Germany; e Martin-Luther-University Halle-Wittenberg, Institute for Medical Epidemiology, Biometry, and Informatics, Halle, Germany; f Martin-Luther-University Halle-Wittenberg, Digital Research Methods in Medicine Group, Halle, Germany; g Martin-Luther-University Halle-Wittenberg, Biobank of the University Medicine Halle, Halle, Germany

**Keywords:** Albuminuria, Urine albumin-creatinine ratio, Pulse wave velocity, Arterial stiffness, Cardiovascular risk, Epidemiology

## Abstract

**Background:**

Albuminuria even at low levels and increased arterial stiffness are associated with higher cardiovascular risk. Very low-grade albuminuria (VLGA; urinary albumin–creatinine ratio [uACR] <30 mg/g) has been linked to adverse outcomes, but its association with arterial stiffness in the general population, particularly among individuals without major cardiometabolic disease, remains uncertain.

**Methods:**

We analyzed data from 7613 participants of the German National Cohort (NAKO) with available uACR and brachial–ankle pulse wave velocity (PWV[ba]) measurements. Multivariable linear regression was used to assess the association between log₁₀-transformed uACR and PWV(ba), adjusting for age, sex, systolic blood pressure, obesity indices, LDL-cholesterol, HbA1c, and estimated glomerular filtration rate (eGFR). A low-risk subgroup excluded participants with arterial hypertension or diabetes mellitus.

**Results:**

Among included participants, 49.6 % were female and 7100 (93.3 %) had uACR <30 mg/g. In the full cohort, a 10-fold higher uACR was associated with higher PWV(ba) by 0.10 m/s (95 % CI 0.03–0.17). Similar associations were observed in the VLGA subgroup (0.16 m/s; 95 % CI 0.07–0.25). In the low-risk VLGA subgroup (*n* = 4308), uACR remained positively associated with PWV(ba) (0.18 m/s per 10-fold higher uACR; 95 % CI 0.08–0.28).

**Conclusion:**

Higher uACR was consistently associated with greater arterial stiffness across the full cohort and among individuals with very low-grade albuminuria, including those without arterial hypertension or diabetes mellitus.

**Figure:**
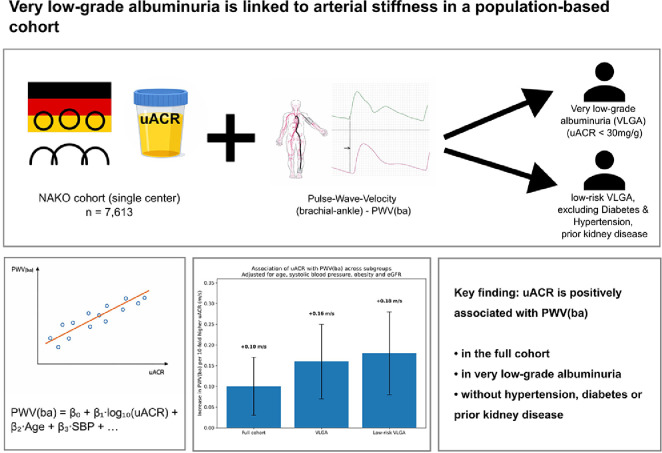
Central Illustration.

## 1 Introduction

Albuminuria in the general population relates to an increased risk of mortality, myocardial infarction, and progression of kidney disease, independent of the initial kidney function [[1], [2], [3], [4]]. Furthermore, albuminuria as a marker of endothelial dysfunction is associated with the progression of coronary heart disease [5] and is in addition linked to increased coronary artery calcification [6]. Albuminuria is also a known marker of progression of diabetic kidney disease [7]. Already very low levels of albuminuria (urinary albumin-creatine-ratio (uACR) < 30 mg/g) are associated with an increased probability of cardiovascular events, hospitalization, or death [8,9]. Microvascular cardiac dysfunction measured as impaired flow-reserve is related to the degree of albuminuria in diabetic individuals, which was also evident in the range of albuminuria levels below 30 mg/g [10]. A recent meta-analysis of over 219,000 participants with a uACR <30 mg/g evaluated the risk of cardiovascular mortality in individuals with uACR levels between 10 and 30 mg/g compared with those with uACR levels below 5 mg/g. This analysis demonstrated a more than twofold increased risk (hazard ratio 2.12; 95 % CI, 1.61–2.80) [11]. These findings indicate that clinically relevant differences in cardiovascular risk exist within the range currently considered normo-albuminuric, suggesting that uACR may capture risk gradients below the conventional 30 mg/g threshold.

Enhanced pulse-wave-velocity (PWV) is a hallmark of vascular stiffness and is associated with an increased number of cardiovascular events in individuals with diabetes mellitus and CKD. It shows promising results as an early diagnostic marker for cardiovascular disease (CVD) in this population [12,13].

Prior studies have reported inconsistent findings regarding the association between uACR and PWV. In the HELIUS study, aortic stiffness was associated with albuminuria only among individuals with type 2 diabetes [14]. A study from China reported an association between uACR and carotid–femoral PWV primarily among individuals with high-normal albuminuria or macroalbuminuria [15]. In contrast, an analysis of 812 participants from the GENOA cohort found no association between aortic PWV and uACR [16]. These discrepancies may reflect differences in study populations, sample sizes, and the distribution of albuminuria levels. Several prior cohorts were enriched for individuals with established cardiometabolic disease or familial cardiovascular risk, and many included relatively small numbers of participants within the very low uACR range, limiting power to detect associations in normoalbuminuric and otherwise low-risk individuals. Thus, it remains uncertain whether subtle elevations of albumin excretion are associated with arterial stiffness in the general population, particularly among individuals without overt cardiovascular disease or diabetes.

Based on these considerations, we hypothesized that very low-grade albuminuria (uACR < 30 mg/g) is independently associated with higher arterial stiffness, assessed by brachial-ankle PWV (PWV(ba)), in the study population. Secondly, we hypothesize that this association is present even among individuals without diabetes, hypertension, or chronic kidney disease, indicating relevance in a cardiovascular low-risk subgroup.

## 2 Methods

### 2.1 Study population

The German National Cohort (NAKO) is a prospective, population-based cohort study that recruited 205,415 participants aged 19 to 74 years from 18 study centers across Germany between 2014 and 2019. The primary aim of NAKO is to investigate the etiology, incidence, and progression of major diseases, with a focus on identifying modifiable risk factors to inform and enhance preventive health strategies. All participants provided written informed consent prior to enrollment. The study protocol was approved by the appropriate institutional ethics committees [17,18]. The present analysis is based on baseline data from a single study center located in Halle (Saale), Germany.

#### 2.1.1 Data collection and handling

Detailed descriptions of the NAKO study design and methodology have been published previously [17,18]. During baseline examination, all participants underwent standardized assessment. Information on smoking history, alcohol consumption, and physical activity was collected using a standardized touchscreen-based questionnaire. Medication use was recorded through a structured face-to-face interview, during which all medications taken within the previous seven days were documented using specialized software. Participants reporting the use of medications classified under Anatomical Therapeutic Chemical (ATC) codes C02, C03, C07, C08, or C09 during this period were categorized as users of antihypertensive drugs.

#### 2.1.2 Pulse wave velocity (PWV) and blood pressure assessment

We measured PWV using the Vascular Explorer system (Enverdis GmbH, Jena, Germany) [19]. Participants rested in a supine position for at least 10 min prior to the assessment to ensure standardized measurement conditions. The device conducted pulse wave analysis and simultaneously recorded ankle and brachial blood pressures using photoplethysmographic signals from the fingers and toes, along with pressure measurements from occlusion cuffs.

To minimize variability, participants were instructed to refrain from speaking and to breathe slowly during the measurement. For the present analysis, PWV(ba) is defined as:

$$PWV\left(ba\right)=\frac{L_{a}-L_{b}}{PTT}$$

where *PTT* denotes pulse transit time (in seconds), *Lₐ* represents the distance from the jugular notch to the ankle cuff (in meters), and Lb the distance from the jugular notch to the brachial cuff (in meters).

Blood pressure was assessed twice on the right arm using the OMRON HEM-705CP oscillometric device (Omron Corporation, Kyoto, Japan) after a rest period of at least 5 min before the first measurement and a break of 2 min between measurements; the second measurement was used for the analysis.

In large population-based studies, brachial–ankle PWV (PWV(ba)) offers practical advantages over carotid–femoral PWV, including shorter examination time and reduced operator dependence, while showing strong correlation with central arterial stiffness and independent associations with cardiovascular outcomes. Accordingly, PWV(ba) is well suited for evaluating vascular aging in epidemiological cohorts [20,21].

Arterial hypertension was defined as systolic blood pressure ≥140 mmHg and/or diastolic blood pressure ≥90 mmHg at examination, self-reported physician diagnosis of hypertension, or current use of antihypertensive medication.

#### 2.1.3 Laboratory measurements

Plasma levels of glycated hemoglobin (HbA1c) were determined using a turbidimetric inhibition immunoassay (TINIA), and serum concentrations of high-density lipoprotein cholesterol (HDL-C) and low-density lipoprotein cholesterol (LDL-C) were measured with homogeneous enzymatic colorimetric assays. Urine albumin was measured using an immunoturbidimetric assay, and serum and urine creatinine were quantified with a kinetic colorimetric assay based on the Jaffé method. All analyses were conducted using a Roche Cobas 8000 modular analyzer (Roche Diagnostics, Rotkreuz, Switzerland) in the Department of Laboratory Medicine, Unit II Laboratory Medicine and Clinical Chemistry of the University Hospital Halle (Saale), according to the manufacturer’s instructions and manuals, with routine maintenance and quality control procedures.

#### 2.1.4 Statistical methods

Descriptive statistics were computed using the tableone R package with stratification for VLGA and albuminuria (AU). VLGA was defined as any uACR below 30 mg/g. Participants with an uACR equal or above 30 mg/g were classified as AU. Skewed variables (e. g., uACR, urinary albumin) were summarized with medians and interquartile ranges. Group differences of median were calculated using the Hodges-Lehmann estimator. Continuous and categorical variables were compared using appropriate non-parametric methods. We used a multivariable linear regression model to assess the association between PWV(ba) and uACR. Covariates were selected a priori based on established associations with both albuminuria and arterial stiffness reported in prior literature. To minimize overadjustment and collider bias, we additionally evaluated a reduced adjustment set informed by conceptual causal diagrams. All analyses were interpreted associative, and no causal inference was intended. We used the software dagitty to construct the graph and to determine the minimal sufficient adjustment set [22]. Consequently, we adjusted for the following covariates: systolic blood pressure (continuous), age, sex, dyslipidemia (LDL-C), obesity indices and diabetes mellitus (HbA1c). All multivariable models were additionally adjusted for estimated glomerular filtration rate (eGFR, continuous) to account for potential confounding by kidney function. Prior to the inclusion in a joint model, we assessed multicollinearity by calculating variance inflation factors. We used the weight-adjusted waist index (WWI), calculated as waist circumference (cm) divided by the square root of body weight (kg), as an anthropometric marker of obesity [23,24]. We verified model assumptions by inspecting residual-versus-fitted, Q–Q, scale–location, and residuals-versus-leverage plots to assess linearity, normality, homoscedasticity, and influential observations. Moreover, no patterns suggesting nonlinearity or the need for alternative modeling approaches were detected. We log-transformed uACR due to extreme right skewness. For urine albumin values below the detection limit of 3 mg/L, we assigned an estimated value of 1.5 mg/L to minimize the risk of over- or underestimating true albuminuria. We conducted a sensitivity analysis to verify this approach. The overall results of the linear regression analysis were not affected after excluding participants with urine albumin below the detection limit (Supplementary Table S1). Linear regression analyses were conducted on the entire cohort and subsequently stratified by VLGA, with further stratification by sex. For analyses in the low-risk subgroup, participants were excluded if they met the study definition of arterial hypertension (systolic blood pressure ≥140 mmHg and/or diastolic blood pressure ≥90 mmHg at examination, self-reported physician diagnosis, or current antihypertensive medication use), had diabetes mellitus (HbA1c ≥ 48mmol/mol, self-reported physician diagnosis), or reported a prior physician diagnosis of impaired kidney function or chronic kidney disease. Pre-existing kidney disease was defined solely by self-report and not by laboratory measurements. All statistical analyses were conducted using R (Version 4.5.2, R Core Team, Vienna, Austria).

## 3 Results

### 3.1 General characteristics

**Fig. 1:**
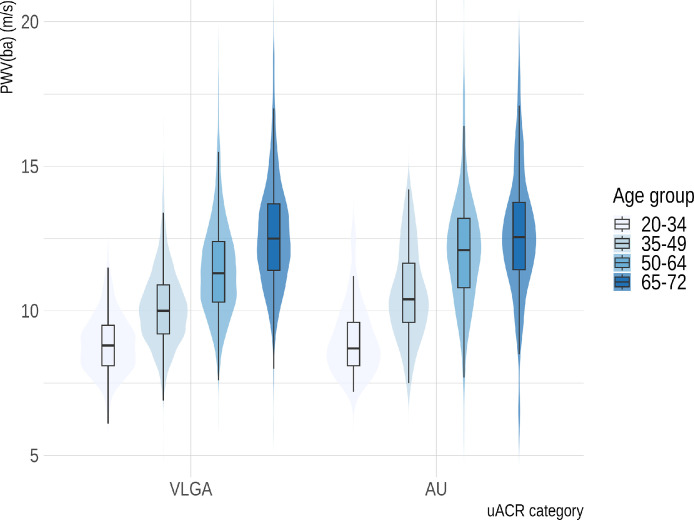
Distribution of brachial-ankle pulse wave velocity (in m/s) by age group and urinary albumin-to-creatinine ratio (uACR) category. Violin plots depict the density distribution, with embedded boxplots showing medians and interquartile ranges across age groups. (VLGA, urine albumin-creatinine ratio (uACR) < 30mg/g) and albuminuric (AU, uACR ≥ 30mg/g) participants. baPWV: brachial-ankle pulse-wave-velocity, VLGA: very low-grade albuminuria.

We analyzed data from 10,139 participants; finally, we included 7613 participants with data on albuminuria and PWV(ba) in the following analyses. The 2526 excluded observations exhibited predominantly similar characteristics as the study population. We excluded observations because of missing data on uACR or PWV. Among the included, 49.6 % were female (Table 1). The median uACR was slightly higher in women than men, with a difference of 1.6 mg/g (95 % CI: 1.3–1.7 mg/g). A total of 7100 participants were classified as having VLGA, while 513 exhibited an uACR ≥ 30 mg/g (AU) (Table 1). Participants in the AU group were, on average, 4.5 years older than those with VLGA. PWV(ba) increased progressively with age across both uACR categories, with the highest values observed among participants aged 65–72 years (Fig. 1). The mean PWV(ba) was slightly higher in the AU group compared with the VLGA group (11.6 ± 2.2 m/s vs. 10.7 ± 1.9 m/s, respectively; Table 1), particularly in older age groups (Fig. 1). The AU group also showed differences in the distribution of comorbidities, with a higher prevalence of diabetes mellitus and arterial hypertension (Table 1). Participants with uACR ≥30 mg/g had a higher treatment intensity, with substantially more frequent use of antihypertensive therapy (51.5 % vs. 30.5 %), ACE inhibitors/ARBs (26.1 % vs. 15.2 %), and statins (16.4 % vs. 7.2 %) compared with those with uACR <30 mg/g (Supplementary Table S2). Despite broadly similar LDL-C levels, this group had higher blood pressure, HbA1c, and PWV(ba), indicating greater vascular burden alongside more intensive pharmacotherapy.

**Table 1:** Baseline demographic and clinical characteristics of participants stratified by albuminuria status. Abbreviations: BP, blood pressure; BMI, body mass index; eGFR, estimated glomerular filtration rate; CKD, chronic kidney disease; uACR, urinary albumin–creatinine ratio; HbA1c, glycated hemoglobin; LDL-C, low-density lipoprotein cholesterol; HDL-C, high-density lipoprotein cholesterol; PWV(ba), brachial–ankle pulse wave velocity; WWI, weight-adjusted waist index. ^1^Smoking status is reported for all participants; pack-years are calculated among ever-smokers only (former and current smokers).

	Overall	VLGA (uACR < 30 mg/g)	AU (uACR ≥ 30 mg/g)
n	7613	7100	513
Age, years (mean (SD))	50.1 (12.6)	49.8 (12.5)	54.3 (12.3)
Female sex (%)	3775 (49.6)	3524 (49.6)	251 (48.9)
Bodyweight, kg (mean (SD))	79.3 (16.7)	79.1 (16.5)	82.2 (19.4)
BP systolic, mmHg (mean (SD))	128.1 (15.9)	127.7 (15.6)	134.7 (18.9)
BP diastolic, mmHg (mean (SD))	78.3 (9.6)	78.1 (9.5)	80.8 (11.1)
WWI (mean (SD))	10.5 (0.8)	10.4 (0.8)	10.8 (0.9)
eGFR, ml/min/1.73m² (mean (SD))	98.4 (15.0)	98.8 (14.6)	93.5 (18.8)
CKD Stage (n, %)			
G1	5520 (73.9)	5178 (74.3)	342 (68.3)
G2	1854 (24.8)	1730 (24.8)	124 (24.8)
G3a	74 (1.0)	53 (0.8)	21 (4.2)
G3b	17 (0.2)	5 (0.1)	12 (2.4)
G4	3 (0.0)	1 (0.0)	2 (0.4)
Urine albumin, mg/l (median (IQR))	3.1 (1.5, 7.9)	3.0 (1.5, 6.4)	57.5 (29.0, 122.6)
uACR, mg/g (median (IQR))	4.0 (1.5, 8.7)	3.6 (1.5, 7.3)	59.8 (41.5, 118.7)
HbA1c, mmol/mol (mean (SD))	35.1 (6.9)	34.8 (6.4)	39.1 (11.2)
LDL-C, mmol/l (mean (SD))	3.4 (1.0)	3.4 (1.0)	3.3 (1.1)
HDL-C, mmol/l (mean (SD))	1.5 (0.4)	1.5 (0.4)	1.4 (0.5)
Cholesterol, mmol/l (mean (SD))	5.2 (1.1)	5.2 (1.1)	5.3 (1.2)
Antihypertensive medication (%)	2428 (31.9)	2164 (30.5)	264 (51.5)
Arterial Hypertension (n, %)	2596 (34.2)	2332 (32.9)	264 (51.9)
Prior kidney disease (n, %)	198 (2.6)	156 (2.2)	42 (8.2)
Diabetes mellitus (n, %)	534 (7.0)	433 (6.1)	101 (19.8)
PWV (ba), m/s (mean (SD))	10.8 (1.9)	10.7 (1.9)	11.6 (2.2)
Smoking status (%)^1^			
Never	3339 (43.9)	3151 (44.4)	188 (36.6)
Former	2282 (30.0)	2135 (30.1)	147 (28.7)
Current	1750 (23.0)	1600 (22.5)	150 (29.2)
Packyears (mean (SD))	13.5 (14.2)	13.2 (14.0)	17.4 (15.6)
Amount pure alcohol consumed, g (mean (SD)) per day	10.4 (16.1)	10.4 (15.9)	11.3 (18.1)

### 3.2 Associations of albuminuria and cardiometabolic factors with pulse-wave-velocity

**Fig. 2:**
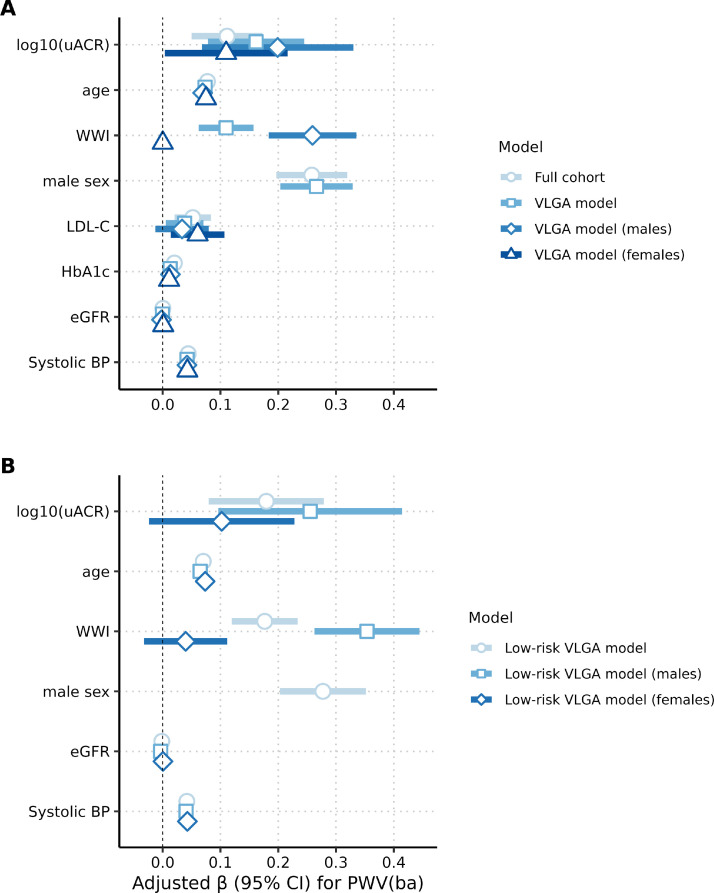
Multivariable-adjusted associations of clinical and metabolic factors with PWV(ba).Panel A shows adjusted β estimates (95 % confidence intervals) for associations with brachial–ankle pulse wave velocity (PWV[ba]) in the full cohort, the very low-grade albuminuria (VLGA) subgroup (uACR <30 mg/g), and sex-stratified VLGA models. Panel B shows corresponding associations in the low-risk VLGA subgroup after exclusion of participants with arterial hypertension, antihypertensive medication use, diabetes mellitus, or self-reported chronic kidney disease, including sex-stratified models. All models were adjusted for age, systolic blood pressure, adiposity (weight-adjusted waist index, WWI), kidney function (estimated glomerular filtration rate, eGFR), lipid levels (LDL-cholesterol), glycemic status (HbA1c), and sex where applicable. Points represent adjusted β coefficients, and horizontal bars represent 95 % confidence intervals. PWV(ba), brachial–ankle pulse wave velocity; uACR, urinary albumin–creatinine ratio; VLGA, very low-grade albuminuria; WWI, weight-adjusted waist index; eGFR, estimated glomerular filtration rate; LDL-C, low-density lipoprotein cholesterol; HbA1c, glycated hemoglobin; BP, blood pressure.

Multivariable regression analyses are summarized in Table 2. In the full cohort, higher urinary albumin–creatinine ratio (log_10_(uACR)) was independently associated with higher PWV(ba), with an estimated increase of 0.10 units per log_10_ increase in uACR (95 % CI: 0.03–0.17). Age and systolic blood pressure showed strong and consistent positive associations with PWV(ba) (Table2, Fig. 2A). Greater weight-adjusted waist index (WWI) was also associated with higher PWV(ba) (β = 0.11, 95 % CI: 0.06–0.16). Male sex (β = 0.24, 95 % CI: 0.18–0.30), LDL-cholesterol (β = 0.04, 95 % CI: 0.00–0.07), and HbA1c (β = 0.01, 95 % CI: 0.01–0.02) were additionally associated with PWV(ba), whereas estimated glomerular filtration rate (eGFR) showed effect estimates close to zero (Table 2, Fig. 2A).

**Table 2:** Results of multivariable linear regression models, including the full cohort and the VLGA subgroup. PWV(ba) as the dependent variable and log_10_-transformed uACR as the independent variable. VLGA: very low-grade albuminuria (uACR < 30mg/g); uACR: urinary albumin-creatinine ratio; WWI: weight-adjusted waist index; LDL-C: low density lipoprotein cholesterol; eGFR: estimated glomerular filtration rate; BP: blood pressure; 95 %CI: 95 % confidence interval (robust).

	Full cohort β-estimate (95 %CI) (*n* = 7613)	VLGA model β-estimate (95 %CI) (*n* = 7100)	VLGA model (males) β-estimate (95 %CI) (*n* = 3576)	VLGA model (females) β-estimate (95 %CI) (*n* = 3524)
log_10_(uACR)	0.10 (0.03, 0.17)	0.16 (0.07, 0.25)	0.20 (0.06, 0.34)	0.11 (0.00, 0.22)
Age (years)	0.07 (0.07, 0.08)	0.07 (0.07, 0.08)	0.07 (0.06, 0.07)	0.07 (0.07, 0.08)
WWI	0.11 (0.06, 0.16)	0.11 (0.06, 0.16)	0.26 (0.18, 0.34)	0.00(−0.06, 0.06)
Sex (male vs. female)	0.24 (0.18, 0.30)	0.27 (0.20, 0.33)	-	-
LDL-C (mmol/l)	0.04 (0.00, 0.07)	0.04 (0.00, 0.07)	0.03 (−0.01, 0.08)	0.06 (0.01, 0.11)
HbA1c (mmol/ mol)	0.01 (0.01, 0.02)	0.01 (0.01, 0.02)	0.01 (0.00, 0.02)	0.01 (0.00, 0.02)
eGFR (ml/min/1.73m²)	0.00 (0.00, 0.00)	0.00 (0.00, 0.00)	0.00 (−0.01, 0.00)	0.00 (0.00, 0.00)
Systolic BP (mmHg)	0.04 (0.04, 0.04)	0.04 (0.04, 0.04)	0.04 (0.04, 0.05)	0.04 (0.04, 0.05)
R^2^	0.55	0.55	0.52	0.57

In the very low-grade albuminuria (VLGA) subgroup, the association between log10(uACR) and PWV(ba) was of similar magnitude (β = 0.16, 95 % CI: 0.07–0.25). Age, systolic blood pressure, WWI, male sex, LDL-cholesterol, and HbA1c remained positively associated with PWV(ba), while eGFR again showed no meaningful association.

Sex-stratified analyses in the VLGA subgroup demonstrated heterogeneity in associations of uACR and WWI with PWV(ba) (Table 2). The association between log_10_(uACR) and PWV(ba) was numerically higher in men (β = 0.20, 95 % CI: 0.06–0.34; R² = 0.52) than in women (β = 0.11, 95 % CI: 0.00–0.22; R² = 0.57). WWI showed a pronounced positive association with PWV(ba) in men (β = 0.26, 95 % CI: 0.18–0.34), whereas the corresponding estimate in women was close to zero (β = 0.00, 95 % CI: −0.06–0.06) (Table 2, Fig. 2A). Age and systolic blood pressure remained consistently associated with PWV(ba) in both sexes, while LDL-cholesterol was positively associated with PWV(ba) in women but not in men. In the VLGA subgroup, the univariable linear regression exhibited a positive association between PWV(ba) and log₁₀(uACR) (β = 0.67, 95 % CI (0.55, 0.79)), explaining 2 % of the variance (R² = 0.02).

To evaluate potential effect modification by sex, interaction terms between sex and log_10_(uACR) were added to the multivariable model in the full cohort (Supplementary Table S3). The interaction estimate for sex and log10(uACR) was small and imprecise (β for interaction = −0.07, 95 % CI: −0.19 to 0.05), indicating little evidence for clinically meaningful sex-related differences in the association between uACR and PWV(ba) after multivariable adjustment. Main associations of age, systolic blood pressure, LDL-cholesterol, and HbA1c with PWV(ba) remained stable, while effect estimates for eGFR were close to zero (Supplementary Table S3).

Because uACR was modeled on the log₁₀ scale, β coefficients represent differences in PWV(ba) per 10-fold higher uACR. In the full cohort, a 10-fold increase in uACR (e.g., from 30 to 300 mg/g) was associated with higher PWV(ba) by 0.10 m/s (95 % CI 0.03–0.17). Corresponding estimates were 0.16 m/s (95 % CI 0.07–0.25) in the VLGA subgroup, 0.20 m/s (95 % CI 0.06–0.34) among men with VLGA, and 0.11 m/s (95 % CI 0.00–0.22) among women with VLGA (Table 2).

We further examined whether the relationship between uACR und PWV(ba) persists in the absence of overt comorbidities. We conducted an additional analysis excluding participants meeting the study definition of arterial hypertension, diabetes mellitus, or pre-existing kidney disease. This low-risk VLGA subgroup (*n* = 4308) was generally slightly younger, had a lower body weight, slightly lower WWI, lower blood pressure and pulse wave velocity. This group also had a better kidney function, as reflected by higher mean eGFR and lower uACR (Supplementary Table S4). In multivariable models within this low-risk subgroup, log_10_(uACR) remained positively associated with PWV(ba) (β = 0.18, 95 % CI: 0.08–0.28), alongside age, systolic blood pressure, WWI, and male sex (Table 3, Fig. 2B). In sex-stratified models, the uACR–PWV(ba) association was larger in men (β = 0.26, 95 % CI: 0.09–0.42; R² = 0.54) than in women (β = 0.10, 95 % CI: −0.03–0.23; R² = 0.59), although estimates in women were less precise. WWI showed a strong positive association with PWV(ba) in men (β = 0.35, 95 % CI: 0.26–0.45) but not in women (β = 0.04, 95 % CI: −0.03–0.11). Across all models, effect estimates for eGFR were close to zero, while systolic blood pressure and age remained consistently associated with PWV(ba) (Table 3, Fig. 2B). In the low-risk VLGA subgroup, a 10-fold increase in uACR (e.g., from 30 to 300 mg/g) was associated with higher PWV(ba) of 0.18 m/s (95 % CI 0.08–0.28) overall, 0.26 m/s (95 % CI 0.09–0.42) in men, and 0.10 m/s (95 % CI −0.03 to 0.23) in women per 10-fold higher uACR (Table 3). Additional adjustment for LDL-cholesterol did not materially affect effect estimates or model fit and was therefore not retained in the final low-risk models. Excluding uACR from the low-risk models did not materially change overall model fit (R² = 0.57 overall, 0.54 in men, and 0.59 in women). Additional adjustment for cardiometabolic medication use did not substantialy change the association between uACR and PWV(ba) (β≈0.10 per log₁₀ increase in uACR in both models), and model fit remained unchanged (R² = 0.55), indicating that the observed association was robust to adjustment for pharmacotherapy (Supplementary Table S5).

**Table 3:** Results of multivariable linear regression models, excluding participants with comorbidities (arterial hypertension, antihypertensive medication use, diabetes mellitus, or pre-existing kidney disease). PWV(ba) as the dependent variable and log10-transformed uACR as the independent variable. VLGA: very low-grade albuminuria (uACR < 30mg/g); uACR: urinary albumin-creatinine ratio; WWI: weight-adjusted waist index; LDL-C: low density lipoprotein cholesterol; eGFR: estimated glomerular filtration rate; BP: blood pressure; 95 %CI: 95 % confidence interval (robust).

	Low-risk VLGA model β-estimate (95 %CI) (*n* = 4308)	Low-risk VLGA model (males) β-estimate (95 %CI) (*n* = 2118)	Low-risk VLGA models (females) β-estimate (95 %CI) (*n* = 2190)
log_10_(uACR)	0.18 (0.08, 0.28)	0.26 (0.09, 0.42)	0.10 (−0.03, 0.23)
Age (years)	0.07 (0.07, 0.07)	0.06 (0.06, 0.07)	0.07 (0.07, 0.08)
WWI	0.18 (0.12, 0.24)	0.35 (0.26, 0.45)	0.04 (−0.03, 0.11)
Sex (male vs. female)	0.28 (0.20, 0.35)	-	-
eGFR (ml/min/1.73m²)	−0.00 (−0.00, 0.00)	−0.00 (−0.01, 0.00)	0.00 (−0.00, 0.00)
Systolic BP (mmHg)	0.04 (0.04, 0.04)	0.04 (0.04, 0.05)	0.04 (0.04, 0.05)
R^2^	0.57	0.54	0.59

Consistent with the interaction analyses in the full cohort, interaction terms between sex and log_10_(uACR) and between sex and WWI were added to the multivariable model in the low-risk subgroup. The interaction estimate for sex and log_10_(uACR) was small with a wide confidence intervall (β for interaction = 0.13, 95 % CI: −0.07 to 0.33), indicating little evidence for meaningful sex-related differences in the association between uACR and PWV(ba) (Supplementary Table S6). In contrast, the interaction between sex and WWI was larger and more precisely estimated (β for interaction = 0.24, 95 % CI: 0.14 to 0.34) (Supplementary Table S6), consistent with a stronger association between central adiposity and PWV(ba) in men compared with women, mirroring the pattern observed in the full cohort and VLGA subgroup.

## 4 Discussion

We observed a positive association between PWV(ba) and log_10_(uACR) in a large population-based cohort study. This finding was present for males and females and persisted after excluding participants with a uACR ≥ 30 mg/g. Moreover, this finding was persistent after exclusion of participants with known comorbidities (arterial hypertension, diabetes mellitus or known kidney disease). This observation held true for both sexes, although sex-stratified analyses suggested numerically larger uACR–PWV associations in men, formal interaction models did not support meaningful effect modification by sex. Such discrepancies may reflect reduced precision after stratification and differences in covariate distributions between men and women.

The minimal change in model R² after inclusion of uACR indicates that traditional determinants such as age and blood pressure dominate cross-sectional variability in PWV(ba). However, the persistence of an independent association suggests that very low-grade albuminuria may capture early vascular vulnerability not fully reflected by conventional risk factors. In relative terms, the association between uACR and PWV(ba) was smaller than that observed for systolic blood pressure, a well-established determinant of arterial stiffness. In our models, systolic blood pressure was associated with approximately 0.04 m/s higher PWV(ba) per 1 mmHg increase (0.40 m/s per 10 mmHg), whereas a 10-fold higher uACR was associated with approximately 0.10–0.18 m/s higher PWV(ba), depending on the subgroup analyzed. This difference in magnitude is expected given the dominant role of blood pressure in arterial stiffening.“Importantly, the stability of effect estimates after additional adjustment for cardiometabolic medication classes argues against confounding by treatment intensity and supports an independent association between albuminuria and arterial stiffness; however, as several medications may lie on the causal pathway between cardiometabolic risk and vascular damage, these analyses were considered sensitivity analyses rather than primary causal models.

### 4.1 Risk of atherosclerotic vascular disease in cohorts with VLGA

An association between VLGA and atherosclerosis was described in a recent cohort study that enrolled 1756 men with an eGFR >60 mL/min/1.73 m² and uACR in the range of 10.0–29.8 mg/g [25]. Participants were recruited while a routine occupational health examination was carried out. The study population was comparable to ours, except for the fact that only men were recruited. The authors concluded that high-normal albuminuria is associated with increased intima-media thickness (IMT) and increased carotid plaque number. In another publication using data from the Cardiovascular Health Study (CHS), which is a population-based cohort study of individuals aged ≥65 years from the US (2006), a relation between microalbuminuria (defined as uACR of 30–300 mg/g) and subclinical atherosclerosis (IMT > 80th percentile, ABI 〈 0.9 and/ or left ventricular mass 〉 80th percentile) could not be confirmed. However, the study found an association between overt cardiovascular disease and microalbuminuria [26]. Another prospective cohort study from Shanghai, China which included over 9500 participants, reported an adjusted odds-ratio (OR) of 1.15 (95 %CI 1.02 to 1.30) for subclinical atherosclerosis in the VLGA group [2]. Further, Liu and colleagues report a substantially increased risk for manifest atherosclerosis of the carotid artery (adjusted OR: 1.22 (95 %CI 1.07 to 1.38)). Similar results yielded the Changfeng Study, in which a positive association between IMT and VLGA was reported [27]. In a meta-analysis of 14 studies including 105,872 participants, Matsushita and colleagues demonstrated a linear increase in cardiovascular and all-cause mortality beginning at a uACR of 5 mg/g, independent of kidney function [8]. A separate meta-analysis stratifying uACR into three categories (<5 mg/g, 5–10 mg/g, and 10–30 mg/g) reported a twofold higher risk of cardiovascular mortality in the highest group compared with the lowest (HR: 2.12; 95 % CI: 1.61–2.80), with even intermediate levels (5–10 mg/g) associated with increased risk (HR: 1.50; 95 % CI: 1.14–1.99) [11]. These findings question the traditional definition of “normo-albuminuria” as uACR <30 mg/g, since cardiovascular risk is already elevated at lower levels.

### 4.2 Pulse-Wave-Velocity and VLGA

McEniery and colleagues found that global endothelial function correlates with central and (somewhat less) peripheral pulse pressure and is independently and inversely correlated with aortic PWV [28]. Endothelial dysfunction of the small vessels is associated with increased albuminuria in prevalent CKD patients [29]. Moreover, albuminuria is considered a surrogate parameter for endothelial dysfunction in individuals with diabetic kidney disease (DKD) [30]. Cross-sectional and prospective data suggest a bidirectional relationship between PWV and uACR. In a Chinese cohort of 1343 participants, carotid-femoral PWV(cf) was in positive linear relation to uACR (β = 0.093) analyzed for high-normal albuminuria (defined as uACR 6.36–30 mg/g) [15]. Consistent with our analysis, the authors performed a multivariable linear regression adjusted for systolic blood pressure, age, uric acid, heart rate, and fasting plasma glucose. They found the strongest association for PWV and uACR in normotensive, elderly males. This corresponds with our results. In another large cross-sectional study of over 5600 participants from a community in Beijing, the investigators found that PWV(ba) showed a stronger association with uACR than PWV(cf) using an adjusted logistic model (odds ratio PWV(ba) 1.06; 95 %CI, 1.03 to 1.10). The authors conclude that PWV(ba) holds strong potential for large-scale community health screening [31]. A cross-sectional analysis of data from the Jackson Heart Study showed a higher prevalence of high-normal uACR (> 25 mg/g in men and >35 mg/g in women) in those with increased PWV(cf) (OR 1.66 (95 %CI 1.32 to 2.11)) among African Americans [32]. Comparable results were reported from the Framingham Offspring study, in which an elevated uACR (uACR ≥17 mg/g (men) or ≥25 mg/g (women)) was linked to elevated PWV(cf) (1.28 (1.02–1.61)) [33]. Furthermore, Vasan and colleagues observed that greater arterial stiffness was associated with a higher prevalence and incidence of target organ damage (albuminuria, left ventricular hypertrophy, covert brain infarcts) and served as a predictor of cardiovascular events [33].

In the GENOA study, Coutinho et al. investigated the relationship between arterial stiffness and markers of subclinical target-organ damage in a community-based cohort enriched for hypertension and reported that aortic (carotid–femoral) PWV was associated with several cardiovascular damage markers but not independently associated with urinary albumin excretion after multivariable adjustment [16]. Differences in arterial stiffness measures may also contribute to the discrepant findings between studies. GENOA assessed carotid–femoral PWV as a measure of central elastic artery stiffness, whereas we used brachial–ankle PWV, which incorporates both central and peripheral arterial segments and yields systematically higher absolute values that are not directly comparable to cfPWV [34]. Peripheral muscular arteries may be particularly sensitive to early endothelial and microvascular dysfunction, which could enhance detection of associations with very low-grade albuminuria. In addition, cohort-specific differences in age distribution, adiposity, and measurement protocols may influence absolute PWV levels. Thus, differences in PWV methodology and population characteristics likely contribute to variation in both mean PWV values and observed associations with albuminuria. In a longitudinal study (5.3 years) of 629 elderly Icelandic adults, researchers investigated the relationship between arterial stiffness and kidney function, focusing on urinary albumin-to-creatinine ratio (uACR) and estimated glomerular filtration rate (eGFR). It is remarkable that despite associations between PWV(cf) with augmentation index on eGFR decline, none of the vascular tonometry measures were associated with changes in uACR [35].

Although in our analysis the regression coefficient for uACR was numerically larger in the low-risk subgroup, this should not be interpreted as evidence of a stronger causal relationship. In cross-sectional analyses, effect estimates may vary across subgroups due to differences in variance structure, competing determinants of PWV, and residual confounding, rather than reflecting true differences in biological effect. In general, a potential association between very low-grade albuminuria and arterial stiffness may reflect shared vascular pathology rather than a direct causal relationship. Subtle increases in urinary albumin excretion may indicate endothelial dysfunction, leading to increased microvascular permeability [36]. Low-grade systemic inflammation may further contribute to both phenotypes, as inflammatory markers such as C-reactive protein are associated with higher uACR and increased PWV, and inflammation promotes arterial elastin degradation, collagen remodeling, and increased glomerular basement membrane permeability [37,38]. Hemodynamic mechanisms may also play a role, whereby increased stiffness of large arteries enhances transmission of pulsatile pressure to the renal microcirculation, particularly in juxtamedullary nephrons with limited autoregulatory capacity [39]. Finally, structural vascular remodeling, including basement membrane thickening and early intima–media changes, may occur in parallel in microvascular and macrovascular beds, supporting the concept that very low-grade albuminuria reflects early, systemic vascular vulnerability rather than isolated kidney involvement [38,39].

### 4.3 Effect of obesity and sex on pulse-wave velocity and albuminuria

There is reliable data that obesity is independently associated with albuminuria [[40], [41], [42]]. Nevertheless, there are things to consider about the interpretation of the uACR in relation to obesity [43,44]. An analysis of data from the large German Study of Health in Pomerania (SHIP) reported a U-shaped association between uACR and obesity indices, including waist circumference and waist-to-height ratio. Interestingly, BMI in this analysis was associated with uACR only at the lower end of the BMI spectrum, whereas higher BMI levels showed no association with uACR [45]. This behavior of BMI in relation to urine creatinine was described for several cohort studies [43,44]. It leads to an underestimation of uACR in higher BMI groups. We therefore substituted BMI by the weight-adjusted waist index (WWI) in our models. The WWI is superior in cardiovascular risk assessment compared to the BMI in individuals with diabetes [46] and in a normal risk cohort [47]. The WWI showed its best performance in CVD risk prediction, compared to BMI, WC, waist-to-height ratio and body shape index [23]. The influence of obesity on PWV was investigated in a French cohort study (*n* = 2304), in which Desamericq and colleagues found no association [48]. In a Korean single center study, involving 3850 participants, waist-to-hip ratio and visceral fat area using bioimpedance, but not BMI and waist circumference, showed an association with PWV [49]. We formally tested for sex interactions and found a significant WWI×sex interaction, indicating a stronger association between WWI and PWV(ba) in men than in women (Supplementary Table S3 and S6). In the interaction model, the WWI×male term showed a precise positive association (β = 0.18, 95 % CI 0.11–0.26), whereas the main WWI effect was attenuated after accounting for sex. This supports the presumption of true effect modification rather than confounding. This finding aligns with prior evidence that WWI is strongly linked to arterial stiffness measured by brachial–ankle PWV. In a recent study of patients with type 2 diabetes, each standard deviation increase in WWI was associated with an approximately 49 cm/s higher baPWV after multivariable adjustment, and WWI showed the strongest correlation with baPWV among several anthropometric indices (*r* = 0.33), outperforming BMI and waist circumference [50]. Sex-specific differences in fat distribution and muscle mass, which WWI is designed to capture, may partly explain the stronger association in men, whereas higher relative fat mass and lower creatinine excretion in women may reduce contrast across WWI levels. Together, these data support WWI as a relevant marker of arterial stiffness, with evidence of sex-dependent effect size.

A large meta-analysis of 167 studies (*n* = 509,743) reported sex related differences in PWV. Lu and colleagues stated that males had a markedly faster increase of PWV(ba) than females during young adulthood, but sex differences vanished at higher ages [51]. Interestingly, they further report a substantially higher age-standardized PWV(ba) in Asia compared to Europe (+1.83 m/s) [51]. In a study from Finland on healthy young volunteers (*n* = 799), a higher PWV was seen in males (8.9 ± 1,8 m/s) than in females (8.1 ± 2.0 m/s), although using a different measurement technique (whole-body impedance cardiography) [52]. Doonan et al. found similar sex-specific differences (men: 6.0 ± 0.7 m/s, women: 5.6 ± 0.6 m/s) in healthy young individuals (*n* = 112) [53]. This observation was reflected in our results. An additional consideration is that uACR depends on urinary creatinine excretion, which differs by sex. Lower creatinine excretion in women can shift the uACR distribution upward even at low absolute albumin excretion, potentially affecting comparability at very low uACR levels and attenuating associations in sex-stratified analyses [54].

### 4.4 Strengths and limitations

This study leverages a large, population-based cohort, enhancing the external validity and applicability of its findings. Participants are comprehensively characterized across demographic, clinical, and lifestyle domains, enabling rigorous adjustment for confounding and detailed subgroup analyses. Importantly, the cohort reflects the characteristics of the general cardiovascular risk in Europe, increasing the relevance of these findings to similar populations and informing preventive strategies. To our knowledge, no prior study has demonstrated an association between very low-grade albuminuria and pulse wave velocity in a low-risk group at the population level.

Several limitations should be acknowledged. The sample was derived from one center within the NAKO cohort, which may introduce some degree of regional bias. However, the large sample size and population-based recruitment likely mitigate major threats to generalizability. Arterial stiffness was assessed using brachial–ankle PWV rather than carotid–femoral PWV, the reference standard for central arterial stiffness, and absolute values are therefore not directly comparable across studies. Urinary albumin excretion was assessed from a single spot urine sample, which is subject to considerable day-to-day variability and may introduce random misclassification, likely biasing associations toward the null. Detailed information on smoking intensity at the time of assessment and dietary protein intake, both of which can influence albumin excretion, was not taken into account. Finally, the cross-sectional design precludes causal inference and does not allow assessment of prognostic implications, which will require longitudinal analyses within the NAKO cohort.

### 4.5 Conclusion

We observed a consistent positive association between PWV(ba) and uACR in the overall cohort and within the very low-grade albuminuria (VLGA) subgroup, which remained evident after exclusion of participants with major cardiometabolic comorbidities. These findings suggest that variations in albumin excretion well below the conventional threshold for albuminuria are associated with concurrent differences in arterial stiffness and may reflect early, shared vascular vulnerability rather than isolated kidney involvement.

Future longitudinal studies are needed to determine whether very low-grade albuminuria and arterial stiffness provide incremental prognostic information beyond established cardiovascular risk factors and whether they may be useful for characterizing early vascular vulnerability in preventive settings.

## Disclosures

MG, CG, OT, AK, TS, AW, BLK, BK, RM, and MZ declare that they have no conflicts of interest to disclose. MGir reports lecture fees from Amgen, Astellas, Bayer Vital, Daiichi. Sankyo, Hexal, Sanofi, Vifor, outside the submitted work; He is engaged in complimentary work for the Commission for Hygiene and Infection Prevention, German Society of Nephrology and as Member of the Board of the German Society for Applied Hygiene in Dialysis. VW received a lecture fee from Bayer Pharmaceuticals and received financial support for congress costs from Lilly Pharma Germany.

## Funding

The German National Cohort (NAKO) is funded by the Bundesministerium für Bildung und Forschung of the Federal Republic of Germany.

## Data sharing statement*

Original data can be requested from the NAKO transfer office. NAKO ensures that the same dataset used in this study will be made available via the transfer office in order to comply with good scientific practice.

## Author declaration

We wish to confirm that there are no known conflicts of interest associated with this publication and there has been no significant financial support for this work that could have influenced its outcome.

We confirm that the manuscript has been read and approved by all named authors and that there are no other persons who satisfied the criteria for authorship but are not listed.

We further confirm that the order of authors listed in the manuscript has been approved by all of us. We confirm that we have given due consideration to the protection of intellectual property associated with this work and that there are no impediments to publication, including the timing of publication, with respect to intellectual property. In so doing we confirm that we have followed the regulations of our institutions concerning intellectual property.

We understand that the Corresponding Author is the sole contact for the Editorial process (including Editorial Manager and direct communications with the office).

He is responsible for communicating with the other authors about progress, submissions of revisions and final approval of proofs.

We confirm that we have provided a current, correct email address which is accessible by the Corresponding Author.

## CRediT authorship contribution statement

**Victor Walendy:** Writing – original draft, Visualization, Validation, Methodology, Formal analysis, Conceptualization. **Michael Gekle:** Writing – review & editing, Data curation, Conceptualization. **Claudia Grossmann:** Writing – review & editing, Data curation. **Alexander Kluttig:** Writing – review & editing, Formal analysis, Data curation. **Frank Bernhard Kraus:** Writing – review & editing, Data curation. **Beatrice Ludwig-Kraus:** Writing – review & editing, Data curation. **Rafael Mikolajczyk:** Writing – review & editing, Formal analysis. **Thomas Schmid:** Writing – review & editing, Formal analysis. **Oliver Thews:** Writing – review & editing, Formal analysis. **Andreas Wienke:** Writing – review & editing, Formal analysis. **Melanie Zinkhan:** Writing – review & editing, Formal analysis. **Matthias Girndt:** Writing – review & editing, Methodology, Formal analysis, Data curation, Conceptualization.

## Declaration of competing interest

The authors declare the following financial interests/personal relationships which may be considered as potential competing interests:

Victor Walendy reports a relationship with Eli Lilly and Company, Bayer Pharmaceuticals Germany that includes: speaking and lecture fees. Matthias Girndt reports a relationship with Amgen Europe GmbH, Astellas, Bayer Vital, Daiichi. Sankyo, Hexal, Sanofi, Vifor,German Society of Nephrology that includes: board membership and speaking and lecture fees. If there are other authors, they declare that they have no known competing financial interests or personal relationships that could have appeared to influence the work reported in this paper.

## Appendix Supplementary materials

Image, application 1
