# Supplementary material for: Very low-grade albuminuria is linked to arterial stiffness in a population-based cohort
Source: Am J Prev Cardiol. 2026 Feb 21;26:101495. doi: 10.1016/j.ajpc.2026.101495 (PMC13083028; doi:10.1016/j.ajpc.2026.101495)
Supplement: Supplementary file 1 [file mmc1.docx]

# Supplement

|  | Full cohort | LGA model | LGA model (males) | LGA models (females) |
| --- | --- | --- | --- | --- |
| Log_10_(uACR) | 0.11 (0.01, 0.21) | 0.27 (0.12, 0.42) | 0.29 (0.07, 0.51) | 0.18 (-0.02, 0.39) |
| Age (years) | 0.07 (0.07, 0.08) | 0.07 (0.07, 0.08) | 0.07 (0.06, 0.08) | 0.07 (0.07, 0.08) |
| WWI | 0.13 (0.08, 0.19) | 0.13 (0.08, 0.19) | 0.28 (0.19, 0.37) | 0.02 (-0.05, 0.09) |
| Systolic BP (mmHg) | 0.04 (0.04, 0.04) | 0.04 (0.04, 0.04) | 0.04 (0.04, 0.05) | 0.04 (0.04, 0.04) |
| LDL-C (mmol/l) | 0.05 (0.01, 0.09) | 0.05 (0.01, 0.09) | 0.05 (-0.01, 0.10) | 0.08 (0.02, 0.14) |
| HbA1c (mmol/mol) | 0.01 (0.00, 0.02) | 0.01 (0.00, 0.02) | 0.01 (0.00, 0.03) | 0.01 (-0.00, 0.02) |
| Sex (male vs. female) | 0.24 (0.16, 0.31) | 0.29 (0.21, 0.36) |  |  |
| eGFR (ml/min/1.73m²) | 0.00 (-0.00, 0.00) | 0.00 (-0.00, 0.00) | 0.00 (-0.00, 0.01) | -0.00 (-0.00, 0.00) |
| R² | 0.54 | 0.55 | 0.52 | 0.56 |

**Supplement Table S1.** Results of multivariable linear regression models examining the association between log₁₀-transformed urinary albumin–creatinine ratio (uACR) and brachial–ankle pulse wave velocity (PWV(ba)) after exclusion of participants with albuminuria values below the detection limit (<3.0 mg/L). Models are shown for the full cohort, the very low-grade albuminuria (VLGA) subgroup (uACR <30 mg/g), and sex-stratified VLGA models. PWV(ba) was the dependent variable in all analyses. Estimates are β coefficients with robust 95% confidence intervals.

Covariates included age, weight-adjusted waist index (WWI), systolic blood pressure, LDL-cholesterol, HbA1c, sex (in non–sex-stratified models), and estimated glomerular filtration rate (eGFR).

Abbreviations: uACR, urinary albumin–creatinine ratio; PWV(ba), brachial–ankle pulse wave velocity; VLGA, very low-grade albuminuria; WWI, weight-adjusted waist index; LDL-C, low-density lipoprotein cholesterol; eGFR, estimated glomerular filtration rate; BP, blood pressure; 95% CI, 95% confidence interval.

|  | Overall | VLGA (uACR < 30 mg/g) | AU (uACR ≥ 30 mg/g) |
| --- | --- | --- | --- |
| n | 7,613 | 7,100 | 513 |
| Age, years (mean (SD)) | 50.1 (12.6) | 49.8 (12.5) | 54.3 (12.3) |
| Female sex (%) | 3775 (49.6) | 3524 (49.6) | 251 (48.9) |
| Bodyweight, kg (mean (SD)) | 79.3 (16.7) | 79.1 (16.5) | 82.2 (19.4) |
| BP systolic, mmHg (mean (SD)) | 128.1 (15.9) | 127.7 (15.6) | 134.7 (18.9) |
| HbA1c (mean (SD)) | 35.13 (6.88) | 34.84 (6.37) | 39.08 (11.19) |
| LDL-C, mmol/l (mean (SD)) | 3.4 (1.0) | 3.4 (1.0) | 3.3 (1.1) |
| HDL-C, mmol/l (mean (SD)) | 1.5 (0.4) | 1.5 (0.4) | 1.4 (0.5) |
| Cholesterol, mmol/l (mean (SD)) | 5.2 (1.1) | 5.2 (1.1) | 5.3 (1.2) |
| Antihypertensive medication (%) | 2428 (31.9) | 2164 (30.5) | 264 (51.5) |
| Arterial Hypertension (n, %) | 2596 (34.2) | 2332 (32.9) | 264 (51.9) |
| Prior kidney disease (n, %) | 198 (2.6) | 156 (2.2) | 42 (8.2) |
| Diabetes mellitus (n, %) | 534 (7.0) | 433 (6.1) | 101 (19.8) |
| PWV (ba), m/s (mean (SD)) | 10.8 (1.9) | 10.7 (1.9) | 11.6 (2.2) |
| ACE-Inhibitor (%) | 671 ( 8.8) | 610 ( 8.6) | 61 (11.9) |
| ATII-Receptor blocker(%) | 538 ( 7.1) | 465 ( 6.6) | 73 (14.2) |
| SGLT2-Inhibtor (%) | 32 ( 0.4) | 29 ( 0.4) | 3 ( 0.6) |
| MRA (%) | 40 ( 0.5) | 36 ( 0.5) | 4 ( 0.8) |
| Statin (%) | 595 ( 7.8) | 511 ( 7.2) | 84 (16.4) |
| GLP1-Receptor agonist (%) | 19 ( 0.3) | 17 ( 0.2) | 2 ( 0.4) |

**Supplementary Table S2.** Baseline characteristics and cardiometabolic medication use stratified by very low-grade albuminuria (VLGA; uACR <30 mg/g) and albuminuria (AU; uACR ≥30 mg/g). Values are presented as mean (SD) or n (%). The table summarizes demographic factors, cardiometabolic risk profile, arterial stiffness (PWV[ba]), and use of antihypertensive, lipid-lowering, and glucose-lowering therapies at baseline.

|  | Interaction model (VLGA subgroup) |
| --- | --- |
| Log_10_(uACR) | 0.12 (0.04, 0.21) |
| Sex (male vs. Female) | -1.62 (-2.41, -0.83) |
| Age (years) | 0.07 (0.07, 0.08) |
| WWI | 0.04 (-0.02, 0.09) |
| Systolic BP (mmHg) | 0.04 (0.04, 0.05) |
| LDL-C (mmol/l) | 0.05 (0.01, 0.08) |
| HbA1c (mmol/mol) | 0.01 (0.01, 0.02) |
| eGFR (ml/min/1.73 m²) | 0.00 (-0.00, 0.00) |
| Log_10_(uACR):sexm | -0.07 (-0.21, 0.06) |
| sexm:WWI | 0.18 (0.11, 0.26) |
| R² | 0.55 |

**Supplement Table S3.** Multivariable linear regression model including interaction terms between sex and log₁₀-transformed urinary albumin–creatinine ratio (uACR) and between sex and weight-adjusted waist index (WWI) in the VLGA subgroup. Brachial–ankle pulse wave velocity (PWV(ba)) was the dependent variable. The model includes main effects for log₁₀(uACR), sex, age, WWI, systolic blood pressure, LDL-cholesterol, HbA1c, and estimated glomerular filtration rate (eGFR), as well as interaction terms for sex×log₁₀(uACR) and sex×WWI. Estimates are β coefficients with robust 95% confidence intervals.

Abbreviations: uACR, urinary albumin–creatinine ratio; PWV(ba), brachial–ankle pulse wave velocity; WWI, weight-adjusted waist index; LDL-C, low-density lipoprotein cholesterol; eGFR, estimated glomerular filtration rate; BP, blood pressure; 95% CI, 95% confidence interval; VLGA, very-ow-grade albuminuria.

|  | Low-risk VLGA subgroup |
| --- | --- |
| n | 4,308 |
| Age at baseline (mean (SD)) | 45.8 (12.5) |
| Sex = female (%) | 2190 (50.8) |
| Weight (kg, mean (SD)) | 75.63 (15.1) |
| Systolic BP (mmHg, mean (SD)) | 125.0 (14.9) |
| Diastolic BP (mmHg, mean (SD)) | 77.2 (9.4) |
| eGFR (CKD-EPI 2021, mean (SD)) | 101.9 (14.1) |
| uACR in mg/g (median (IQR)) | 3.50 (1.50, 7.35) |
| Albuminuria in mg/l (median (IQR)) | 3.00 (1.50, 6.62) |
| PWV(ba) in m/s (mean (SD)) | 10.3 (1.7) |
| Smoking status (%) |  |
| never | 1990 (46.2) |
| former | 1133 (26.3) |
| current | 1079 (25.1) |
| Packyears (mean (SD)) | 11.8 (12.3) |
| WWI (mean (SD)) | 10.2 (0.7) |

**Supplemental Table S4**: General characteristics of the low-risk group (excluding participants with comorbidities: arterial hypertension, antihypertensive medication use, diabetes mellitus, or pre-existing kidney disease). BP: blood pressure; BMI: body mass index; eGFR: estimated glomerular filtration rate; CKD: chronic kidney disease; uACR: urinary albumin-creatinine ratio; VLGA: very low-grade albuminuria (uACR < 30mg/g); PWV(ba): brachial-ankle pulse-wave-velocity; WWI: weight-adjusted waist index

|  | LGA model (medication) | LGA model (medication class) |
| --- | --- | --- |
| age | 0.07 (0.07, 0.08) | 0.07 (0.07, 0.08) |
| WWI | 0.11 (0.06, 0.16) | 0.11 (0.06, 0.16) |
| log10(uACR) | 0.10 (0.03, 0.17) | 0.10 (0.04, 0.17) |
| male sex | 0.24 (0.18, 0.31) | 0.24 (0.18, 0.31) |
| eGFR | 0.00 (-0.00, 0.00) | 0.00 (-0.00, 0.00) |
| Systolic BP | 0.04 (0.04, 0.05) | 0.04 (0.04, 0.05) |
| ACE Inhibitors | -0.07 (-0.20, 0.07) |  |
| Angiotensin II Receptor Blockers | 0.05 (-0.08, 0.19) |  |
| Sodium-glucose co-transporter 2 inhibitors | 0.53 (-0.03, 1.10) |  |
| mineralocorticoid receptor antagonists | 0.05 (-0.41, 0.52) |  |
| statins | -0.02 (-0.18, 0.13) |  |
| Glucagon-like peptide-1 (GLP-1) analogues | -0.01 (-0.47, 0.45) |  |
| Groups |  |  |
| RAAS-i |  | -0.01 (-0.11, 0.09) |
| SGLT2-i/GLP-1RA |  | 0.40 (-0.01, 0.82) |
| Statin |  | -0.03 (-0.18, 0.13) |
| N | 7,379 | 7,379 |
| R2 | 0.55 | 0.55 |
| Standard errors are heteroskedasticity robust. p < 0.001; p < 0.01; p < 0.05. | | |

**Supplementary Table S5.** Sensitivity analyses of the association between very low-grade albuminuria (log₁₀[uACR]) and arterial stiffness (PWV[ba]) with additional adjustment for cardiometabolic medication use. Two multivariable OLS models are shown: one including individual drug classes (ACE inhibitors, angiotensin II receptor blockers, SGLT2 inhibitors, mineralocorticoid receptor antagonists, statins, and GLP-1 receptor agonists) and one including grouped medication classes (RAAS inhibitors, SGLT2 inhibitors/GLP-1 receptor agonists, and statins). Estimates are regression coefficients with 95% confidence intervals; standard errors are heteroskedasticity-robust. Both models demonstrate stable effect estimates for uACR and comparable model fit (R² = 0.55), indicating robustness of the uACR–PWV(ba) association to adjustment for treatment intensity.

|  | Interaction model (VLGA low-risk subgroup) |
| --- | --- |
| Log_10_(uACR) | 0.11 (0.02, 0.23) |
| Sex (male vs. Female) | -2.30 (-3.33, -1.27) |
| Age (years) | 0.07 (0.06, 0.07) |
| WWI | 0.04 (-0.03, 0.11) |
| Systolic BP (mmHg) | 0.04 (0.04, 0.04) |
| LDL-C (mmol/l) | 0.03 (-0.01, 0.08) |
| HbA1c (mmol/mol) | 0.02 (0.00, 0.03) |
| eGFR (ml/min/1.73 m²) | 0.00I (-0.00, 0.00) |
| Log_10_(uACR):sexm | 0.13 (-0.08, 0.34) |
| sexm:WWI | 0.24 (0.14, 0.35) |
| R² | 0.58 |

**Supplemental Table S5.** Multivariable linear regression model including interaction terms between sex and log₁₀-transformed urinary albumin–creatinine ratio (uACR) and between sex and weight-adjusted waist index (WWI) in the low-risk cohort VLGA. Brachial–ankle pulse wave velocity (PWV(ba)) was the dependent variable. The model includes main effects for log₁₀(uACR), sex, age, WWI, systolic blood pressure, LDL-cholesterol, HbA1c, and estimated glomerular filtration rate (eGFR), as well as interaction terms for sex×log₁₀(uACR) and sex×WWI. Estimates are β coefficients with robust 95% confidence intervals.

Abbreviations: uACR, urinary albumin–creatinine ratio; PWV(ba), brachial–ankle pulse wave velocity; WWI, weight-adjusted waist index; LDL-C, low-density lipoprotein cholesterol; eGFR, estimated glomerular filtration rate; BP, blood pressure; 95% CI, 95% confidence interval; VLGA, very-ow-grade albuminuria.
